# Blood transcriptome comparison between sexes and their function in 4-week Rhode Island red chickens

**DOI:** 10.1080/19768354.2022.2146187

**Published:** 2022-11-29

**Authors:** Hana Kim, Hyojun Choo, Jihye Cha, Myoungjin Jang, Juhwan Son, Taejoon Jeong, Bong-Hwan Choi, Youngjo Lim, Han-Ha Chai, Jungjae Lee, Dajeong Lim, Donghyun Shin, Woncheoul Park, Jong-Eun Park

**Affiliations:** a Division of Animal Genomics and Bioinformatics, National Institute of Animal Science, Wanju, Korea; b Poultry Research Institute, National Institute of Animal Science, Pyeongchang, Korea; c Department of Animal Science and Technology, College of Biotechnology and Natural Resources, Chung-Ang University, Anseong, Korea; d Department of Agricultural Convergence Technology, Jeonbuk National University, Jeonju, Korea; e Department of Animal Biotechnology, College of Applied Life Science, Jeju National University, Jeju-si, Korea

**Keywords:** blood, transcriptome, sex, chicken, TGF-beta signaling pathway

## Abstract

Sex is a major biological factor in the development and physiology of a sexual reproductive organism, and its role in the growing process is needed to be investigated in various species. We compare blood transcriptome between 5 males and 5 females in 4-week-old Rhode Island Red chickens and perform functional annotation of differentially expressed genes (DEGs). The results are as follows. 141 and 109 DEGs were located in autosomes and sex chromosomes, respectively. The gene ontology (GO) terms are significantly (*p* < 0.05) enriched, which were limb development, inner ear development, positive regulation of dendrite development, the KEGG pathway the TGF-beta signaling pathway, and melanogenesis (*p* < 0.05). These pathways are related to morphological maintenance and growth of the tissues. In addition, the *SMAD2W* and the *BMP5* were involved in the TGF-beta signaling pathway, and both play an important role in maintaining tissue development. The major DEGs related to the development of neurons and synapses include the up-regulated *NRN1*, *GDF10*, *SLC1A1*, *BMP5*, *NBEA,* and *NRXN1*. Also, 7 DEGs were validated using RT-qPCR with high correlation (*r*^2^ = 0.74). In conclusion, the differential expression of blood tissue in the early growing chicken was enriched in TGF-beta signaling and related to the development of neurons and synapses including *SMAD2W* and *BMP5*. These results suggest that blood in the early growing stage is differentially affected in tissue development, nervous system, and pigmentation by sex. For future research, experimental characterization of DEGs and a holistic investigation of various tissues and growth stages will be required.

## Introduction

Sex is an important factor for growth and physiological status. Poultry is included in the factor (Chamruspollert et al. 2002). In previous studies, differences in the growth of tissues were observed between sexes, and these differences were also detected as carcass, fatty acids, glucose, cholesterol, etc. in physiological and blood parameter analysis (Rondelli et al. 2003; Salim et al. 2012). In addition, sexes and environmental conditions show that females are exposed to aggression and male can indicate copulation-solicitation displays during the breeding season, different behavioral aspects such as hormone and endocrine responses by differential gene expression. The steroid hormone in serum is shown differences between sexes (Gahr 2001; Wang Y et al. 2019). Also, biological characteristics in poultry are regulated through gene expression. Some genes are differentially expressed between sexes, and these genes are overexpressed in sex chromosomes (Brothers et al. 2019). Besides, the growth stages of chickens are generally constituted with early growth stage (4–16-weeks), late growth stage (by 24-weeks), and mature growth stage (after 24-weeks), which can be a factor in phenotype, physiological parameters, and genes expression with sexes. These are reported to be effective before the mature growth stage (Podisi et al. 2013; Li et al. 2017; Xue et al. 2017; Ono et al. 2022). Especially, the early growth stage is expressed various gens related growth for lipid metabolism and hormone regulation in adipose and muscle tissues, and sexes are led to factors affecting differentially expressed genes at this point (Dridi et al. 2007; Abdullah et al. 2010; Ons et al. 2010; Yu-Jiang et al. 2020). However, previous studies were performed using entrails, However, blood was not used in most studies.

In poultry, females have ZW sex chromosomes and males have ZZ sex chromosomes for sex determination, and thus, poultry chromosomes are different from mammals (Bellott et al. 2017). Some animals were females heterogamety as ZW and highly expressed genes in the W chromosome appeared to have evolved from autosomes, which were identified through gene assembly in previous studies (Nam and Ellegren 2008). Also, the Z chromosome was contrasted with the Y chromosome of mammals and was commonly studied to understand the sex determination process in chickens (Storchová and Divina 2006). Among the identified genes, *HINTW* (Histidine triad nucleotide binding protein W), *HNRNPKL* (Heterogeneous nuclear ribonucleoprotein K-like), *SMAD2W* (SMAD family member 2 W), and *SPINW* (Spindlin W) are homologous genes in the W chromosome, related to growth and development of tissues in the ovary (Ceplitis and Ellegren 2004; Kaiser and Ellegren 2006; Mishra et al. 2020). Especially, homologous genes from the W chromosome were overexpressed in female-specific tissue as the ovary, which could inhibit homologous genes of the Z chromosome. These genes are observed with a higher gene expression than autosome genes (Bellott et al. 2017). The W chromosome is typically smaller than the Z chromosome, and at least 70% includes repetitive sequences that inhibit the completion of chromosome assembly. So, such genes are not completely mapped, and little is known about their gene expression. (Tomaszkiewicz et al. 2017). In provided studies, transcriptomic comparison between sexes is focused on detecting sex-biased gene and related sex chromosome, whereas compared studies using normal gene and their roles with autosomes is not common. Especially, such studies were not reported in chicken blood of early growth stage, are reported in mammals, and are not completely determined with poultry blood (Jansen et al. 2014; Ramstad et al. 2016; Wang J et al. 2021). The muscle and gonad tissues were used in provided studies (Bai et al. 2020; He et al. 2020).

Therefore, we investigate differentially expressed genes and their roles between males and females through transcriptome analysis using RNA-sequencing in blood from 4-week-old Rhode Island Red chickens. We contribute to research by understanding their genes and metabolic pathways through gene functional analysis in female chickens.

## Materials and methods

### Animal experiments

In animal experiments, we reared localized Rhode Island Red chickens of a pure line from a pedigree selection. All animals were vaccinated according to the vaccine program of the poultry institute, NIAS. These animals were housed in battery cages (0.6728 m^2^/cell, 0.034 m^2^/bird). The animals were divided into male and female groups, and the animals had ad libitum access to water and feed according to the feed program for 4 weeks (CP 19%, ME 2850 kcal). For RNA-sequencing and RT-qPCR (Real-time Quantification PCR), 1–2 ml blood samples were drawn through the wing vein from 5 males and 5 females, and stored in an RNAlater (Thermo Fisher Scientific, Carlsbad, CA, USA) on −20°C. The animal experiment and sacrifice followed ethical guidelines in Poultry research at the National Institute of Animal Science (NIAS20191550).

### RNA isolation and sequencing

For the isolation of RNA, we used the column method following the manufacturer's protocol in each 1 ml blood sample. First, the stored blood samples were centrifuged at 13,000 rpm at 4°C for 20 m, and obtained RNA pellets from centrifuged blood samples. Next, the RNA pellet was purified utilizing ReliaPrep™ RNA Miniprep Systems (Promega, Madison, Wisconsin, USA), and purified RNA was used for cDNA synthesis with SuperScript™ III First-Strand Synthesis System (Thermo Fisher Scientific, Carlsbad, CA, USA) and RNA-sequencing. We synthesized RNA to cDNA was stored at −20°C for RT-qPCR. Then, isolated RNA was measured for integrity and purity through a 2100 Bioanalyzer and RNA Nano 6000 Assay Kit (Agilent Technologies, Santa Clara, CA, USA). The RNA samples identified RIN (RNA integrity number) values, and > 8 were used for the library construction of the random fragmentation of cDNA. The library was constructed using a TruSeq Stranded Total RNA LT Sample Prep Kit (Illumina, San Diego, CA, USA) by following the manufacturer's protocol, sequencing was performed using an Illumina HiSeq 4000 platform with generated paired-end reads. After sequencing, FASTQ raw read files for post-analysis were converted using the bcl2fastq Illumina package from BCL image files.

### Quality control, mapping and counting

For quality control and trimming with < Q20, adapters, and N base in raw reads, we performed FastQC v0.11.5 (released 8 Mar 2016, https://www.bioinformatics.babraham.ac.uk/projects/fastqc) and Trimmomatic v0.39 (Bolger et al. 2014). Then, generated clean reads were mapped to the chicken (GRCg6a.104) reference-based genome using Hisat2 v2.2.1 (Kim et al. 2019). The mapped reads were annotated based on GRCg6a.104 and counted using featureCounts of the Subread package v2.0.1 (Liao et al. 2014). The read counts were identified using a PCA (Principal Component Analysis) plot through a converted log₂+1 value and a calculated PC (Principal Component) value, and we performed DEG (differentially expressed gene) analysis using count files.

### Differentially expressed genes analysis

We determined DEGs between the male and female groups using edgeR package v3.32.0 in R v4.0.3 (Robinson et al. 2010). First, below 10 gene expression counts in the sum of 10 animals were filtered. Thus, the TMM (Trimmed Mean of M-values) method was used for normalization, and estimating dispersions values were acquired. DEGs were considered FDR (False Discovery rate) < 0.05 and 1.0 ± ≤log_2_FC (log_2_ Fold change). Such DEGs were used to draw the heatmap for visualization. Then, DEGs were divided by up-regulated genes and down-regulated genes following the log_2_FC value. Finally, characterized protein-coding genes in localized chromosomes were sorted from divided DEGs for functional analysis.

### Gene ontology, KEGG pathway and network analysis

Enrichment analyses were performed on the determined DEGs using DAVID (Database for Annotation, Visualization, and Integrated Discovery) web tool for identifying gene annotation, molecular function, cellular component, biological processes, and gene pathways with EASE <0.1 (Huang et al. 2009). The selected databases and chicken (gallus gallus) species were GO (Gene Ontology) and KEGG (Kyoto Encyclopedia of Genes and Genomes Pathway) on DAVID (Ashburner et al. 2000; Kanehisa and Goto 2000). Visualization for biological term analysis and functionally grouped network through the ClueGO plugin on GO and KEGG database with *p* < 0.05 were performed on the top pathway genes (Bindea et al. 2009).

### RT-qPCR for validation

We selected 7 genes (7 target genes and 2 reference genes) including Z and W chromosome genes in DEGs, and each sample was performed RT-qPCR through 3-replication. Gene expression patterns were identified for validation of the transcriptomic analysis through RNA-sequencing. A total of 7 target genes were selected according to overexpressed homologous genes, enrichment pathways genes, and randomization. The gene primers provided in sheet data 1 of the Supplementary excel file and the obtained Ct value were calculated using the 2^−ΔΔCt^ method (Livak and Schmittge 2001).

### Statistical analysis

Overall statistical analysis was considered with *p* < 0.05. The physiological parameters were compared in the male and female groups through a t-test. The DEGs were performed following the previous description. The PCA plot, bar plot, and heatmap were performed using ggfortify v0.4.11 (released 3 Oct 2020, github.com/sinhrks/ggfortify), ggplot2 v3.3.3 (released 4 Jan 2021, cran.r-project.org/web/packages/ggplot2/ggplot2.pdf), and pheatmap package v1.0.12 (released 4 Jan 2019, cran.r-project.org/web/packages/pheatmap/pheatmap.pdf) in R for visualization.

## Results

### Construction of the raw reads and mapping

A total of 10 blood samples from the chicken generated raw read count from 62,069,934 to 88,994,230. The Q20 and Q30 percentages were 97.65% to 98.44% and 93.67% to 95.26%. The CG percentages were 43.94% to 45.70%, and the raw reads were mapped at 73.26% to 85.56% to the chicken reference-based genome. Detailed information is provided in sheet data 2 of the Supplementary excel file.

### Identification of DEGs

**Figure 1.**
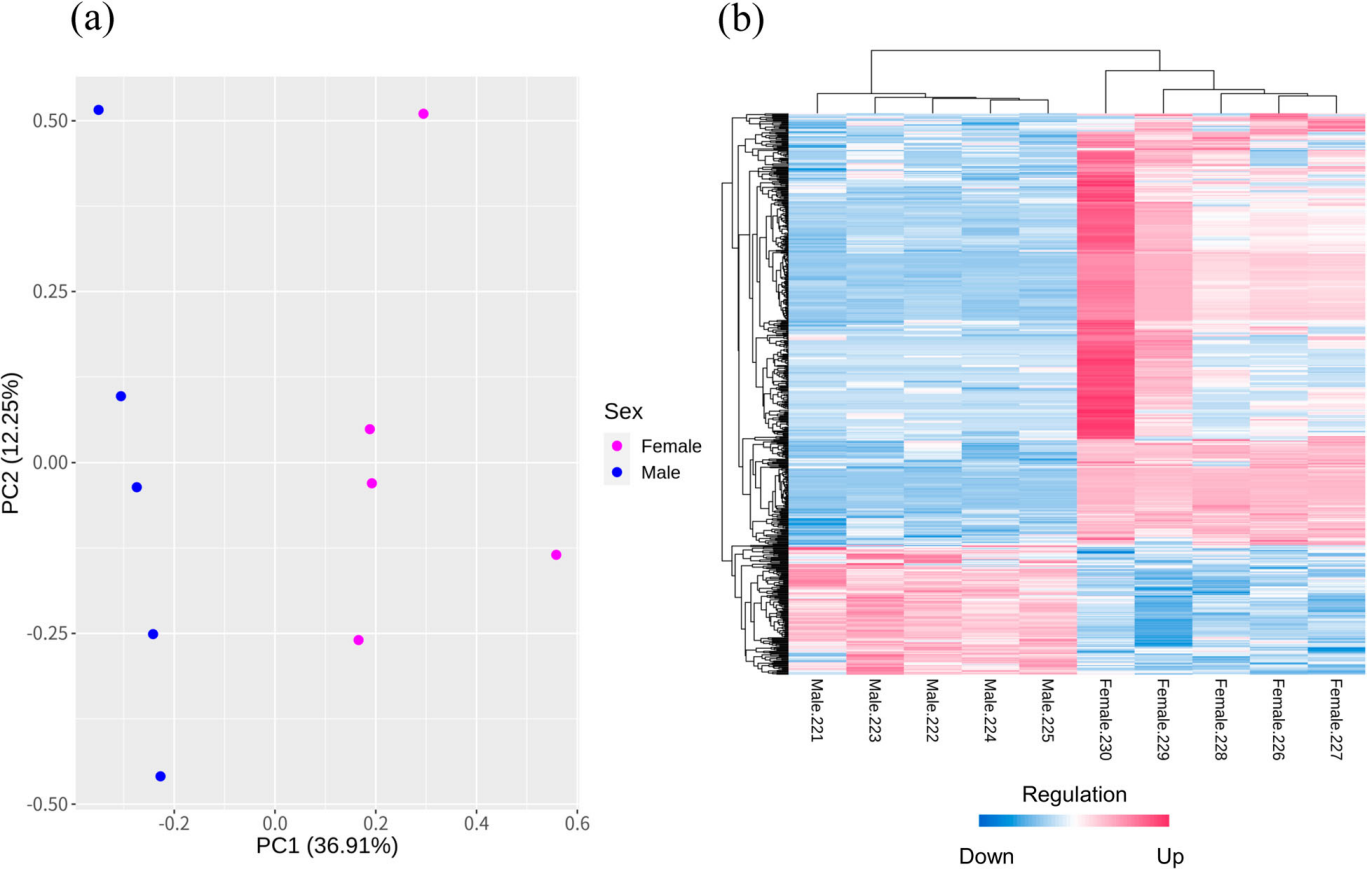
(a) Principal component analysis between Male and Female groups, (b) Heatmap showing the detected DEGs and samples, the magenta and blue colors indicated up-regulated and down-regulated genes, respectively.

**Figure 2.**
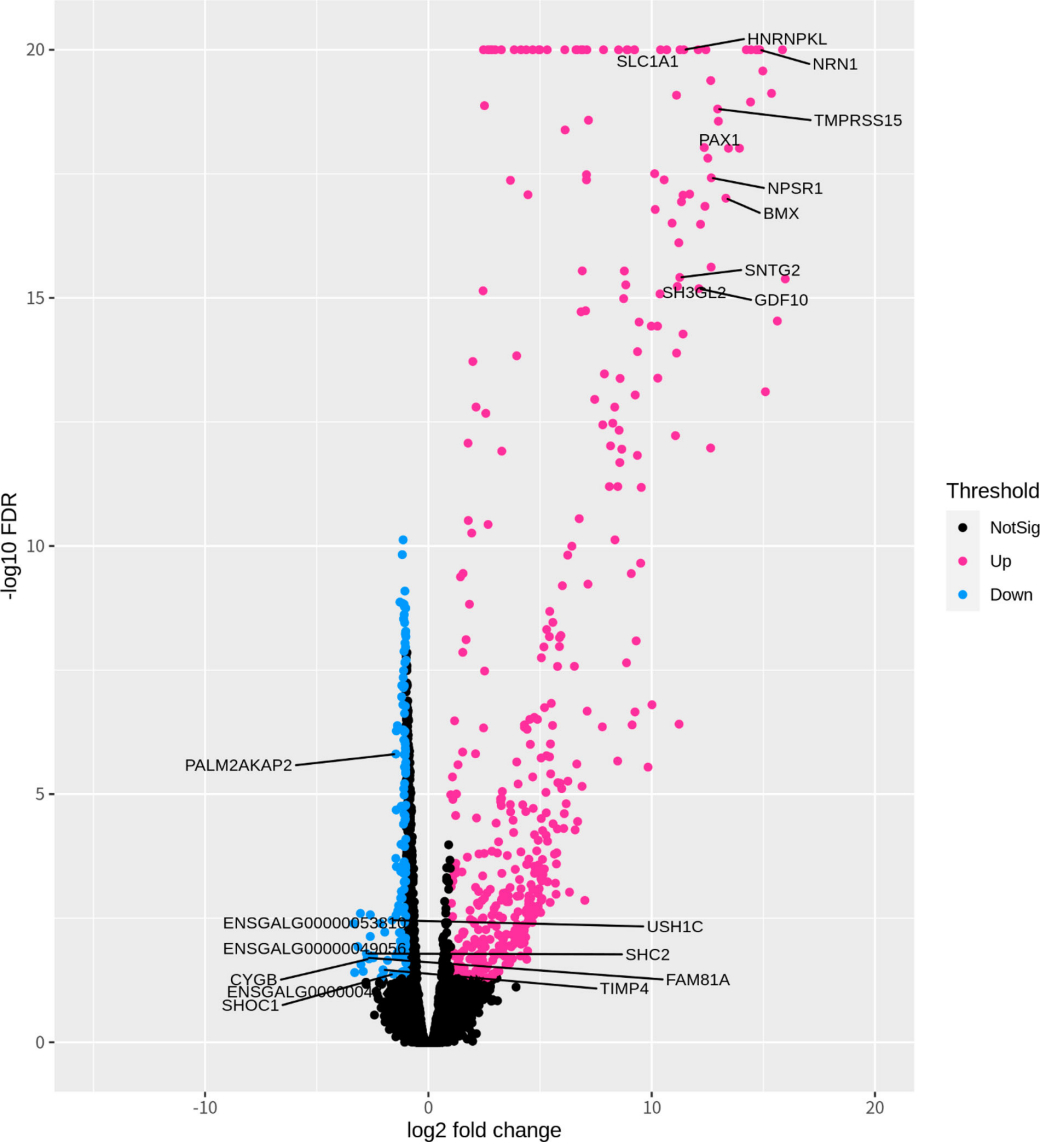
Volcano plot with the top 10 DEGs; each dot color is shown pink as up-regulated and blue as down-regulated

We obtained DEGs with an FDR of <0.05 and 1.0±≤log_2_FC. Out of 24,356 genes, 15,047 genes were identified after filtering. DEGs with the protein-coding gene were 316 constructed genes, and mRNAs with characterized genes were 151 up-regulated genes and 99 down-regulated genes in females. In the chromosomes, the W chromosome had the highest percentage of DEGs, and 54 DEGs were identified out of a total of 86 genes (Table 1 and Figure 1). The top 5 up-regulated DEGs were *NRN1* (Neuritin 1), *BMX* (BMX Non-Receptor Tyrosine Kinase), *TMPRSS15* (Transmembrane Serine Protease 15), *NPSR1* (Neuropeptide S Receptor 1) and *PAX1* (Paired Box 1). The top 5 down-regulated DEGs were *ENSGALG00000053810* (Re-verse transcriptase domain-containing protein), *SHC2* (SHC Adaptor Protein 2), *FAM81A* (Family with Sequence Similarity 81 Member A), *CYGB* (Cytoglobin), and *ENSGALG00000049056* (Reverse transcriptase domain-containing protein). Detailed information on top 10 DEGs can be identified in Figure 2 and sheet data 3 of the Supplementary excel file.

**Table 1.** Percentage of DEGs into chromosomes in females.

Uncharacterized				Characterized			
Chromosome	Genes	DEGs	% of DEGs	DEGs	Regulation		% of DEGs
					Up	Down	
Autosome	14018	132	0.942	109	93	16	0.778
Z	757	115	15.192	105	22	83	13.871
W	86	54	62.791	36	36	0	41.86
MT	13	0	0	0	0	0	0
Unknown	173	15	8.671	4	3	1	2.312
Total	15047	316	2.1	254	154	100	1.688

Z and W: Sex chromosome, MT: Mitochondria.

### Function and pathway analysis of the DEGs.

**Figure 3.**
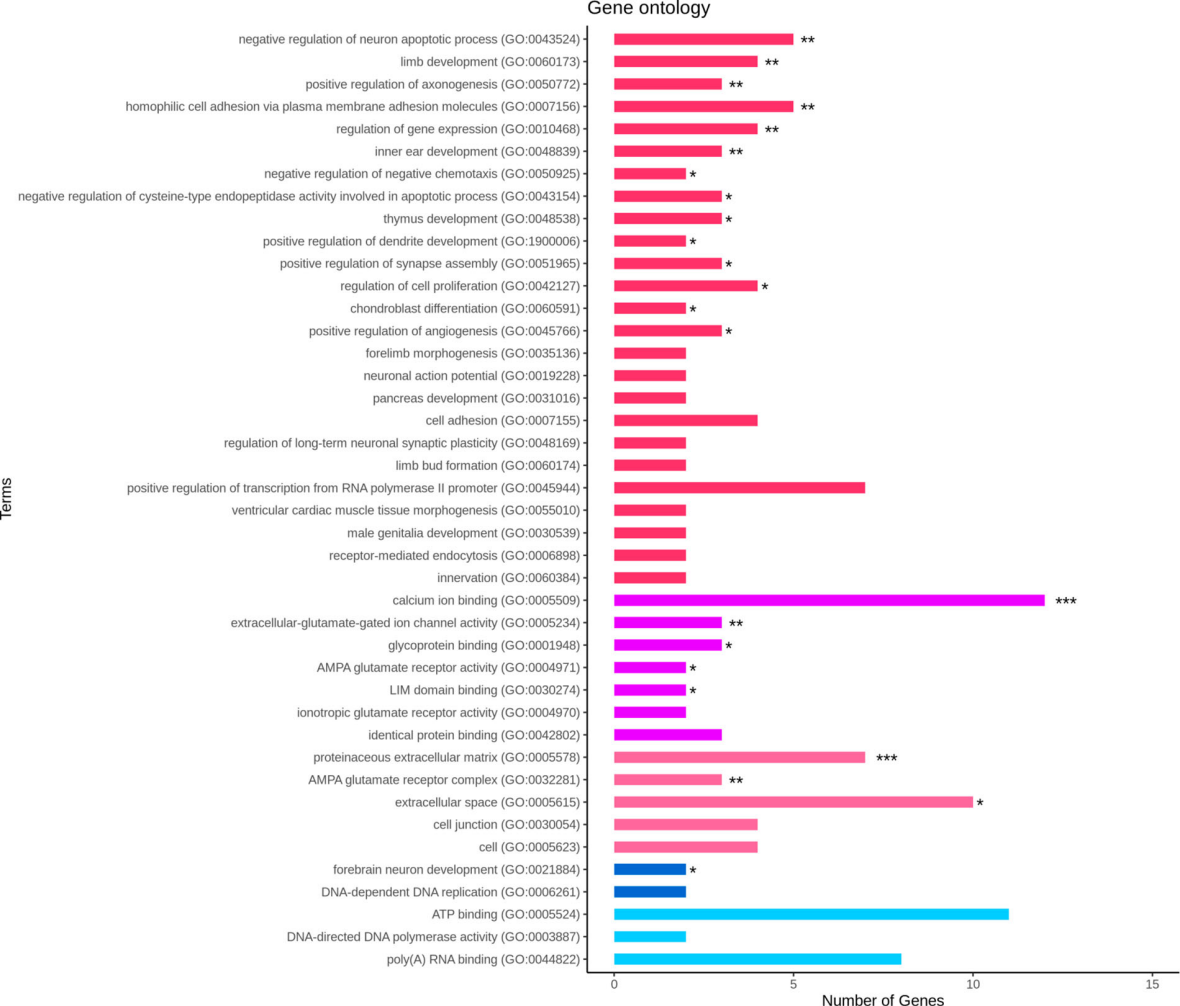
Enriched gene ontology; *p*-value indicated and GO term of up-regulated genes were shown as red (Biological Processes), violet (Molecular Functions) and magenta (Cellular Components) colors; GO term of down-regulated genes were shown as blue (Biological Processes) and cyan (Molecular Functions) colors. * *p* < 0.05, ** *p* < 0.01, *** *p* < 0.001.

**Figure 4.**
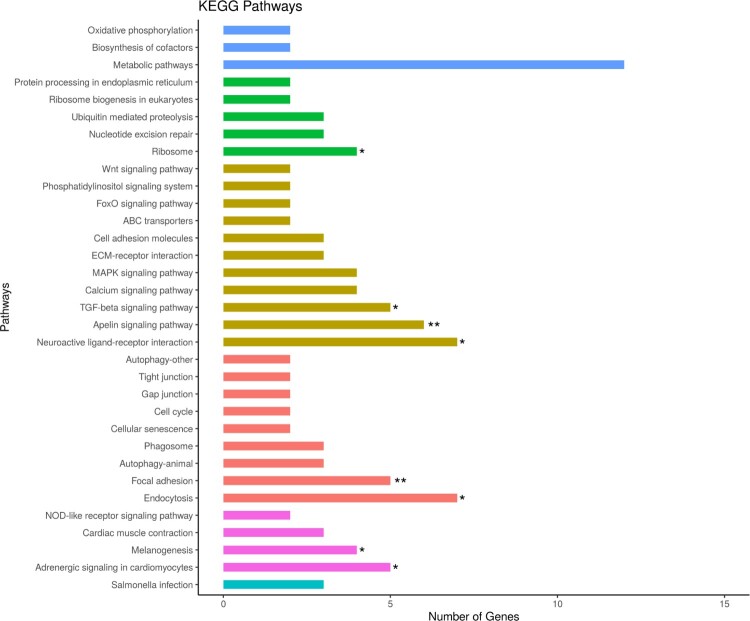
Detected KEGG Pathways; denoted by different colors for the various pathway types and by the number of DEGs. * *p* < 0.05, ** *p* < 0.01.

For functional analysis, the identified 250 DEGs were used for the GO and KEGG pathway analyses. The GO analysis was based on DAVID. We identified 15 biological processes, 3 cellular components, and 5 molecular functions with EASE <0.1 and *p* < 0.05 (Figure 3). The top 10 GO terms were proteinaceous extracellular matrix (GO:0005578), positive regulation of axonogenesis (GO:0050772), limb development (GO:0060173), negative regulation of the neuron apoptotic process (GO:0043524), extracellular-glutamate-gated ion channel activity (GO:0005234), AMPA glutamate receptor complex (GO:0032281), calcium ion binding (GO:0005509), clathrin-coated pit (GO:0005905), homophilic cell adhesion via plasma membrane adhesion molecules (GO:0007156), and negative regulation of negative chemo-taxis (GO:0050925). The KEGG pathway analysis detected a total of 33 pathways (Figure 4). Detailed information on GO terms is provided in sheet data 4 of the Supplementary excel file.

### Network enrichment analysis

**Figure 5.**
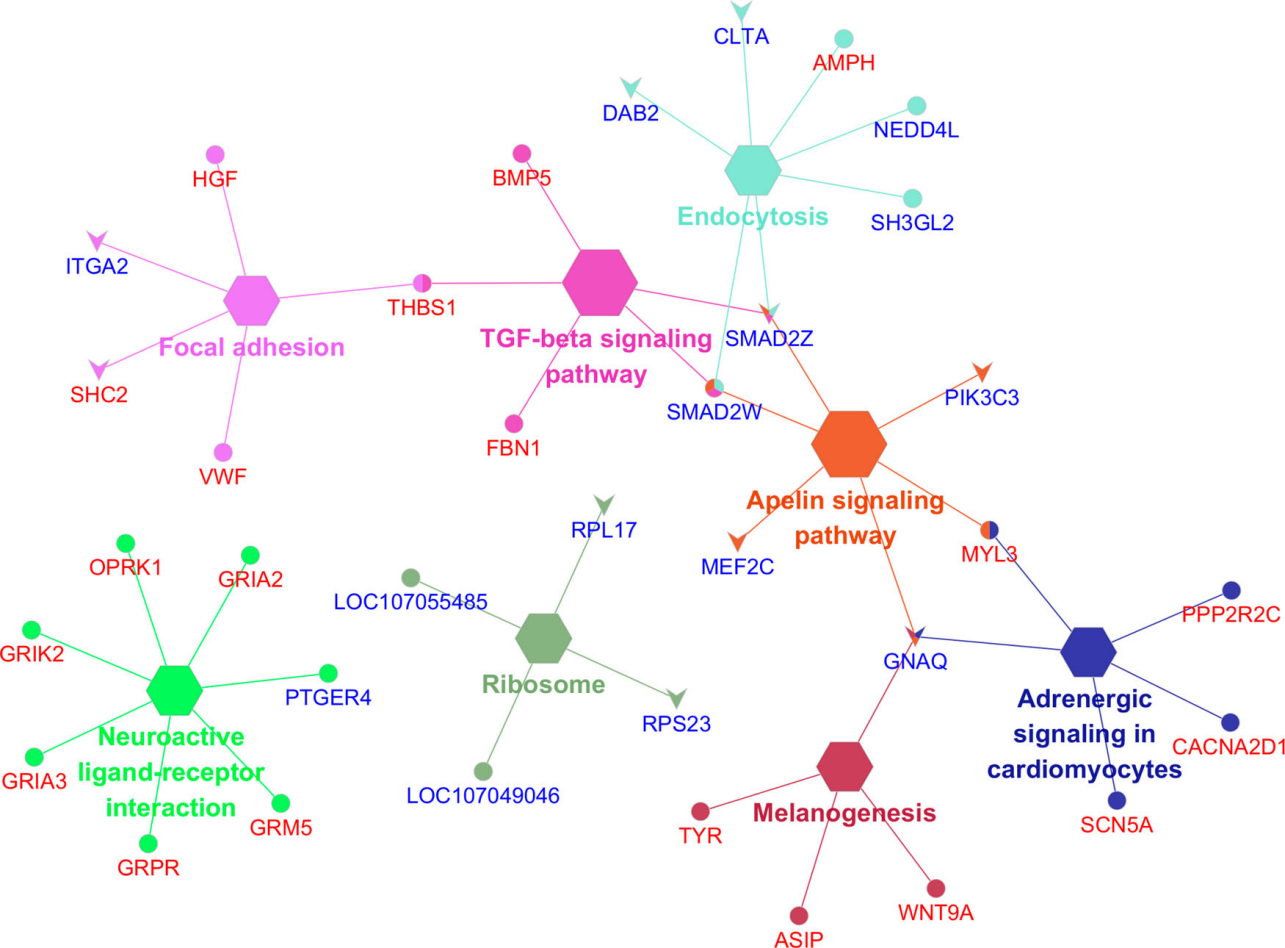
Enriched gene network of the top KEGG pathways analysis with the elliptical shape representing the upregulated genes and the V shape representing the down-regulated genes, red text as the autosome and blue text as the sex chromosome genes, respectively.

From the identified KEGG pathways, we identified the enrichment pathways with *p* < 0.05. The 8 enriched pathways were Endocytosis, Neuroactive ligand–receptor interaction, Apelin signaling pathway, Focal adhesion, Adrenergic signaling in cardiomyocytes, TGF-beta signaling pathway, Melanogenesis, and Ribosome, which are indicated as the gene network in Figure 5. The information of enriched pathways is indicated in sheet data 5 of the Supplementary excel file.

### Validation of DEGs through RT-qPCR

**Figure 6.**
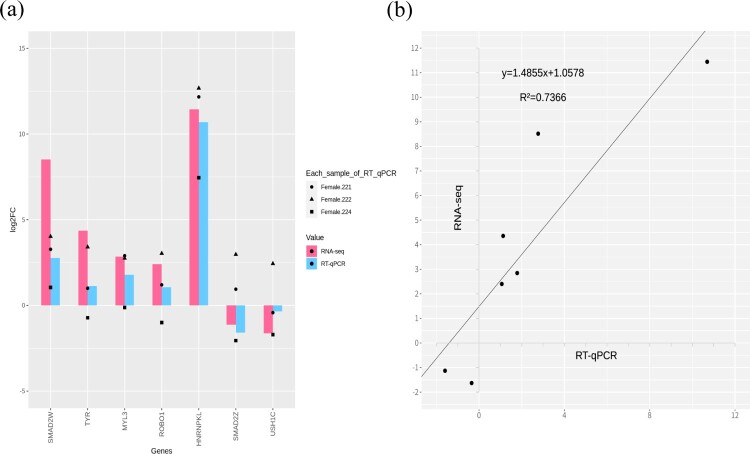
(a) RT-qPCR and RNA-seq results were compared through the bar plot, RNA-sequencing with log_2_FC and RT-qPCR with 2^-ΔΔCt^ indicated the magenta and blue color; (b) Correlation (r^2^) plot of log_2_FC and 2^-ΔΔCt^.

We performed expression validation of DEGs through RT-qPCR, and selected genes such as *HNRNPKL*, *SMAD2W*, *ROBO1* (Roundabout Guidance Receptor 1), *TYR* (Tyrosinase), *MYL3* (myosin light chain 3), *SMAD2Z* (SMAD family member 2 Z), and *USH1C* (USH1 Protein Network Component Harmonin) among the DEGs. As shown in Figure 6, we correlated the results between RNA-sequencing and RT-qPCR with *r^2^*^ ^= 0.7366. The results of RT-qPCR followed the results of RNA-sequencing in up- and down-regulated genes.

## Discussion

In this study, we compared differentially expressed genes between 4-week-old males and females in Rhode Island Red chickens. Among a total of 250 DEGs in females with functional characterization, 105, 36, and 109 genes were located in Z chromosomes, W chromosomes, and autosomes, respectively. In females, a higher percentage of DEGs was observed in Z and W chromosomes than autosomes, characterized DEGs in the Z chromosome were constituted 22 in up-regulated and 83 in down-regulated with 41.86% of DEGs (Table 1). These results are known as dosage compensation mechanisms that occur in heterogametic systems, and such mechanisms are equalized different genes expression by number of sex chromosomes (Julien et al. 2012). Especially, chickens have an incomplete dosage compensation mechanism in Males, is reported that Z chromosome genes in male-derived (ZZ) showed to be more highly expressed than in female-derived (ZW) (Uebbing et al. 2015; Graves 2016). Besides, 36 W chromosome DEGs were identified with 41.86% of DEGs in females. The W chromosome genes of avians had been kept non-transferring between chromosomes including loss of genes, sequence deletions, and repetitive sequence for a large amount of time, and these genes were highly expressed with an unclear number of genes (Ayers et al. 2013; Mishra et al. 2020). Thus, the identified DEGs in sex chromosomes between different sexes should be considered for interpretation. In functional analysis, we detected the enrichment of GO terms, and the KEGG pathway as the Apelin signaling pathway, the melanogenesis, and the adrenergic signaling in cardiomyocytes (*p* < 0.05). These pathways are related to animal growth with *TYR*, *MEF2C*, *SCN5A,* and *MYL3* (Wang Z et al. 2015; D’Mello et al. 2016; Mishra et al. 2020).

The *HNRNPKL*, *HINTW*, *SMAD2W*, *ATP5F1AW* (ATP synthase F1 subunit alpha W), *SPINW*, *LOC100858742* (RNF111_N do-main-containing protein), and *SMAD2Z* were up-regulated in the W chromosome. These DEGs are known as a major role in tissue development (Ceplitis and Ellegren 2004; Rallabandi et al. 2019). Moreover, these DEGs are highly expressed in various other tissues, which means that DEGs are not only related to sex determination. Also, those genes functioned as the indirect development of genital gland tissue in females, DNA binding, gene expression, cell division, and signaling transmission (Amberger et al. 2015). In the Z chromosome, *SLC1A1* (Solute Carrier Family 1 Member 1), *SH3GL2* (SH3 Domain Containing GRB2 Like 2), *RORB* and *RASEF* (RAS and EF-Hand Domain Containing) were up-regulated. These genes had roles in the nervous system, hormone regulation, and GTP binding (Kaiser and Ellegren 2006; Amberger et al. 2015). Functional annotation of DEGs had similar results in the GO term and KEGG pathways analysis. Expressed genes in both sexes were identified as mostly non-sex-specific (Rogers et al. 2021). For example, *SMAD2W* and *SMAD2Z* play roles in the production and growth of tissues, which are regulated differently between sexes. In autosomes, DEGs were *MYL3*, *BMP5*, *NRN1*, *BMX*, *TMPRSS15*, *NPSR1,* and *PAX1*, which are known to perform roles in the nervous system and tissue development (Amberger et al. 2015). In the DEGs, *NRN1*, *GDF10* (Growth Differentiation Factor 10), *SLC1A1*, *BMP5*, *NBEA* (Neurobeachin) and *NRXN1* (Neurexin 1) contribute to the development of neurons and synapse system during the growth stage (Patzke and Ernsberger 2000; Beck et al. 2001; Mecklenburg et al. 2014; Thompson et al. 2014; Zito et al. 2014; Miller et al. 2015). However, such as DEGs, not showing in enrichment analysis, are identified in only DEGs. Moreover, their expression is not described to occur in only females with lacking physiological data. Thus, such results consider that major DEGs are not influenced directly by their role, which can be expressed various genes and reveal hormones related to growth by early growth stage (Jia et al. 2018; Johnsson et al. 2018).

Overall, enrichment pathways were mostly related to growth and development of tissues with GO terms. The 4-weeks old chickens are able to distinguish later sexes with sexual maturity, the identified DEGs and their roles showing that they were growing up through related gene expression (Anh et al. 2015). Among the enrichment pathways, the TGF-beta signaling pathway plays an important role in the growth and maintenance of various tissues with *BMP* (Miyazono 2000; Zuo et al. 2017). Especially, *BMP5* has a role in ovary development by TGF-beta signaling pathway activation (Shimasaki et al. 2004; Divya and Bhattacharya 2021). The *SMAD2* is important in the TGF-beta signaling pathway, which could affect the growth of tissues with the female genital. Thus, the deactivation of the TGF-beta signaling pathway is caused by the reduction of generative functions (Ross and Hill 2008). With BMPs and SMADs, the TGF-beta signaling pathway may be enriched for the growth and development of ovaries, such appears to be related development of various tissues at the growth stage in this result (Zhang et al. 2018; Umair et al. 2020). The activation of the melanogenesis pathway is related to an increase in melanin synthesis. *TYR* (Tyrosinase) in chromosome 1 of chickens is known as an important component of the melanogenesis pathway. *TYR* synthesis of melanin including pigmentation of skin and feathers is affected by androgens in females by the melanogenesis pathway (Yu et al. 2019). The melanogenesis pathway is activated more in males than females, and is known to affect the pigmentation of skin and feathers (Slominski et al. 2004). Therefore, activation of the melanogenesis pathway is associated with the up-regulation of *TYR*, which are Tyrosinase related protein 1 and Tyrosinase related protein 2 genes in avian (D’Mello et al. 2016). In addition, these pathways are involved in the central nervous, circulatory system, growth regulation, cell migration, and signal transduction for growth (Beck et al. 2001). However, environmental factors are not ignored during melamine synthesis, and genetic effects can associate with their pathway activation (Galván and Solano 2016). The sex hormones with estrogen are associated with the activation of the TGF-beta signaling pathway, which is known to activate ovarian growth and cell differentiation with *BMP5* (Shah and Rogers 2018). However, enrichment pathways are not sex-specific in females, because of some of the pathway functions, and the results of significant GO terms and KEGG pathways with DEGs. Also, these results to be identified in only females are hard to describe their meaning, which is needed to elucidate the mechanism of expressed genes changes in different sexes. Moreover, we performed validation of 7 DEGs through RT-qPCR. Seven target genes were identified through a correlation between DEGs analysis and RT-qPCR.

In conclusion, DEGs with W chromosome had the highest percentage in females, and DEGs were involved in various pathways than the DEGs of autosomes. The DEGs were related to tissue growth and the circulatory system in blood. The activation of TGF-beta signaling pathway was activated through up-regulated *SMAD2W* and *BMP5*. Up-regulated *WNT9A*, *ASIP*, and *TYR* activated the melanogenesis pathway and are known to be related to growth and melanin synthesis in the feathers of animals, the circulatory system as well as functions in the maintenance of blood, which are known to be associated with melamine synthesis for pigmentation in skin and feathers. However, most identified pathways are roles for animal growth and melanin with TGF-beta signaling and melanogenesis pathway. There does not appear to be a female-specific role for ovary development in this study. The sex-determination mechanism in avians is not clear, and the composition of the chromosome is not fully known. Such results appear to be shown related to features of expressed genes at the early growth stage, and their mechanism should be more investigated. Thus, some up-regulated genes in the growth stage may function in tissue development and pigmentation. We suggest the need for experimental characterization of DEGs.

## Conclusions

This study identified differentially expressed genes between males and females with pathways in Rhode Island Red breed chickens. The animals are divided into male and female groups of 5 animals each. Hence, transcriptome analysis through RNA-sequencing on the 10 chicken blood samples is performed. The characterized 141 DEGs with sex chromosomes and 109 DEGs with autosomes were identified in the females. In the functional analysis, 23 GO terms were identified, 8 KEGG pathways appear significant, and the pathways involved in tissue development and growth and nervous systems are identified in the early growing stage. Especially, the TGF-beta signaling pathway is activated with the *SMAD2* and *BMP5*, and the melanogenesis pathway with the *TYR*. These pathways are also related to tissue development and the synthesis of melamine for pigmentation in blood. These findings consider that the identified DEGs and their roles showed features of expressed genes at the early growth stage, and further studies are needed for detecting their mechanism. Validation of DEGs is performed through RT-qPCR, and a correlation is identified. Therefore, we suggest that experimental characterization of DEGs is required to investigate various tissues.

## Supplementary Material

Supplemental Material
